# Two-Level Meta-Analysis of Genetic and Epigenetic Markers of Asthma in Preschool Children

**DOI:** 10.3390/jcm15031229

**Published:** 2026-02-04

**Authors:** Snezana Rsovac, Nadja Cukanovic, Luka Zekovic, Vesna Selakovic, Katarina Milosevic

**Affiliations:** 1 Department of Pediatric and Neonatal Intensive Care, University Children’s Hospital, Tirsova 10, 11000 Belgrade, Serbia; 2 Faculty of Medicine, University of Belgrade, Dr Subotića 8, 11000 Belgrade, Serbia; 3 Department of Allergology and Immunology, University Children’s Hospital, Tirsova 10, 11000 Belgrade, Serbia

**Keywords:** asthma, genetic markers, epigenetic markers, meta-analysis

## Abstract

**Background**: Genetic variants within the 17q21 locus and epigenetic modifications regulating immune function have been associated with childhood asthma, yet reported effect sizes vary across studies due to methodological heterogeneity and differences in study design. **Objectives**: To systematically synthesize evidence on genetic and epigenetic markers associated with childhood asthma using a two-level random-effects meta-analysis integrating published meta-analyses and independent cohort studies. **Methods**: PubMed/MEDLINE and Embase were searched for studies published in English between 2011 and 2024. Eligible studies included pediatric populations with asthma or wheeze phenotypes assessing predefined genetic (ORMDL3, GSDMB) or epigenetic (AHRR, FOXP3, CpG loci) markers and reporting odds ratios (ORs) or sufficient data for their derivation. Risk of bias was assessed using established quality criteria for observational studies. Quantitative synthesis was performed using a two-level random-effects model with restricted maximum likelihood estimation. **Results**: Six studies comprising 51,235 children met the inclusion criteria. The overall pooled estimate demonstrated a significant association between molecular markers and childhood asthma (pooled OR = 1.45; 95% confidence interval (CI) 1.30–1.61). Subgroup analyses showed comparable effects for meta-analytic data (OR = 1.39; 95% CI 1.24–1.56) and cohort studies (OR = 1.47; 95% CI 1.31–1.64). Genetic markers yielded a pooled OR of 1.38 (95% CI 1.21–1.56), while epigenetic markers showed a pooled OR of 1.48 (95% CI 1.27–1.73). Heterogeneity in asthma definitions, methylation platforms, and limited representation of non-European populations may affect generalizability. **Conclusions**: This systematic review and two-level meta-analysis provides robust evidence that both genetic and epigenetic variations contribute to childhood asthma susceptibility and supports integrative multi-omic approaches for early-life risk stratification.

## 1. Introduction

Asthma is the most common chronic inflammatory disease of childhood, arising from the interplay between genetic predisposition, epigenetic regulation, and early-life environmental exposures. Despite extensive genome-wide and epigenome-wide association efforts, the molecular mechanisms linking genetic loci and immune dysregulation remain only partially understood. Among the identified loci, the 17q21 region, encompassing ORMDL3 and GSDMB, has consistently shown the strongest association with early-onset asthma across multiple ethnic populations [1,2,3]. Functional studies have demonstrated that ORMDL3 influences endoplasmic reticulum stress and sphingolipid metabolism, thereby amplifying airway inflammation [4,5]. In parallel, epigenetic modifications, particularly methylation changes in AHRR (cg05575921) and FOXP3 promoter regions, modulate T-regulatory cell differentiation and tolerance responses [6,7,8]. Although numerous genome-wide and methylome-wide studies have confirmed these signals, results vary in magnitude and significance due to differences in sample size, phenotype definition, and analytical design. Conventional single-level meta-analyses often ignore the hierarchical nature of accumulated evidence—mixing primary data and prior meta-analyses without accounting for dependence structures. To address these limitations, we performed a two-level random-effects meta-analysis combining both published meta-analyses and independent cohort studies. This hierarchical approach allowed variance partitioning between and within study clusters, providing more reliable estimates of the molecular contribution to asthma risk. Our objective was to quantify the integrated genetic and epigenetic associations with childhood asthma and to evaluate consistency across study designs and biomarker classes.

## 2. Materials and Methods

### 2.1. Protocol and Registration

This systematic review and meta-analysis was conducted in accordance with the PRISMA 2020 guidelines (Supplementary Table S1). A review protocol was not prospectively registered. Given the methodological nature of this hierarchical meta-analysis and the use of previously published aggregate data, protocol registration was not deemed mandatory. No deviations from the planned methodology occurred during the conduct of the review.

### 2.2. Eligibility Criteria

Studies were eligible if they met the following criteria: (i) original cohort studies or published meta-analyses; (ii) pediatric populations with asthma or early wheeze phenotypes (episodic viral wheeze and multitrigger wheeze); (iii) assessment of predefined genetic (ORMDL3, GSDMB) or epigenetic (AHRR, FOXP3, CpG loci) markers; (iv) availability of effect estimates expressed as odds ratios (ORs) with corresponding 95% confidence intervals (CI) or sufficient data for their derivation. Reviews without quantitative synthesis, animal studies, and studies lacking relevant molecular endpoints were excluded.

### 2.3. Information Sources and Search Strategy

A targeted literature search was conducted in October 2025 in PubMed/MEDLINE and Embase to identify relevant studies published from January 2011 up to December 2024. Search terms combined asthma-related keywords with genetic and epigenetic markers. Reference lists of key meta-analyses and reviews were manually screened to ensure completeness. Only peer-reviewed articles published in English were considered.

### 2.4. Study Selection

Titles and abstracts were screened for eligibility, followed by full-text assessment of potentially relevant studies. Study selection was performed independently by two reviewers, with disagreements resolved by consensus.

### 2.5. Data Collection Process

Data extraction was performed independently by two investigators using a predefined extraction form. Extracted variables included study design, population characteristics, molecular markers assessed, sample size, and reported effect estimates.

### 2.6. Risk of Bias Assessment

Risk of bias was assessed qualitatively at the study level based on study design, exposure assessment, outcome definition, and statistical adjustment (Table 1). Only high-quality studies were included.

### 2.7. Effect Measures

The effect measures used for quantitative synthesis were the odds ratio (OR) with corresponding 95% confidence intervals (CIs), reflecting the association between predefined genetic and epigenetic markers and childhood asthma outcomes. When necessary, reported effect estimates were transformed to the natural logarithmic scale prior to analysis. Sampling variances were calculated from published confidence intervals. All effect measures were harmonized to ensure comparability across studies, irrespective of study design or molecular platform.

### 2.8. Synthesis Methods

Quantitative synthesis was performed using a two-level random-effects meta-analysis to account for the hierarchical structure of the evidence base. Previously published meta-analyses were treated as higher-level clusters summarizing multiple primary studies, whereas individual cohort studies were modeled as single-study clusters. Effect estimates were pooled on the logarithmic scale using restricted maximum likelihood (REML) estimation.

The random-effects structure was specified to estimate heterogeneity both between clusters (τ^2^_between) and within clusters (τ^2^_within). This approach allowed partitioning of total heterogeneity into components attributable to differences between meta-analytic evidence and variability among individual studies. Statistical analyses were conducted using the metafor package in R (ver 1.65) (function rma.mv).

Subgroup analyses were performed according to study design (meta-analyses versus cohort studies) and molecular marker category (genetic versus epigenetic). Sensitivity analyses were conducted by excluding smaller studies and by restricting the analysis to high-quality studies only. Statistical heterogeneity was assessed using τ^2^ estimates and variance decomposition, and results were visually presented using forest plots.

### 2.9. Reporting Bias

Publication bias was evaluated using funnel plots and Egger’s regression test.

### 2.10. Certainty

Certainty of evidence was assessed considering consistency, precision, and heterogeneity of results.

## 3. Results

### 3.1. Study Selection

**Figure 1:**
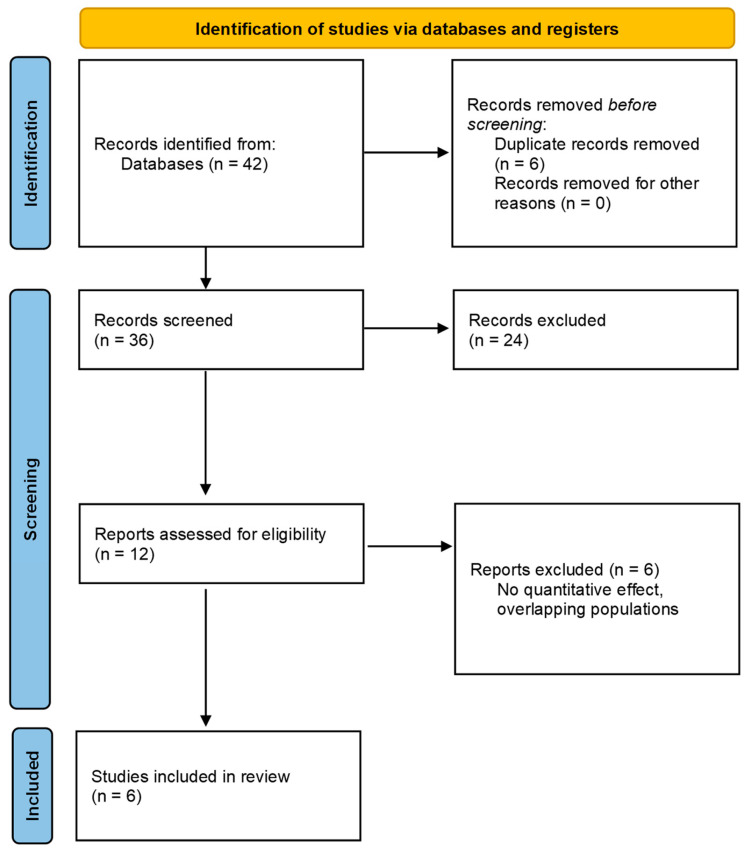
Selection flow diagram.

The systematic search and selection process identified a total of eligible studies investigating genetic and epigenetic determinants of childhood asthma. The search terms used were asthma, genetic markers, epigenetic markers, children, and various combinations thereof. After removal of duplicates and screening of titles and abstracts, full texts were assessed for eligibility. Six quantitative studies met the predefined inclusion criteria and were incorporated into the two-level meta-analysis, comprising two previously published meta-analyses and four independent cohort studies. Together, these studies represented data from 51,235 children. The reasons for exclusion at each stage were predefined and are summarized in the study selection flow diagram (Figure 1).

### 3.2. Study Characteristics

The characteristics of the included studies are summarized in Table 2. The evidence base consisted of two published meta-analyses focusing on genetic variants within the 17q21 locus (ORMDL3, GSDMB), four prospective or population-based cohort studies evaluating epigenetic markers, including AHRR methylation, FOXP3 promoter methylation, and multiple CpG loci.

Sample sizes ranged from 416 to 26,475 participants, and all studies were rated as high methodological quality. Effect estimates were reported as odds ratios (ORs) with corresponding 95% confidence intervals, enabling quantitative synthesis.

### 3.3. Risk of Bias in Included Studies

All included studies were assessed as having a low risk of bias. Meta-analyses were conducted according to established methodological standards, while cohort studies employed validated asthma definitions, standardized genotyping or methylation platforms, and appropriate confounder adjustment. No study was excluded on the basis of quality assessment.

### 3.4. Results of Individual Studies

Across individual studies, all reported effect estimates demonstrated a positive association between molecular markers and childhood asthma. Genetic variants within ORMDL3 and GSDMB yielded odds ratios ranging from 1.33 to 1.45, while epigenetic markers showed ORs between 1.42 and 1.58. No individual study reported a null or protective association (Table 2).

### 3.5. Results of Syntheses

#### 3.5.1. Overall Meta-Analysis

**Figure 2:**
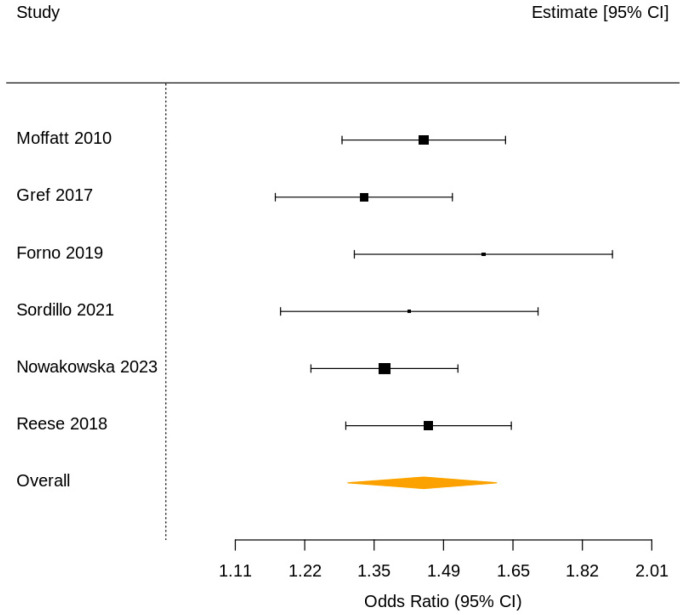
Forest plot—overall. Forest plot showing individual study odds ratios (95% confidence interval (CI)) and the pooled estimate (diamond). All individual study estimates are positioned to the right of the null line (odds ratio (OR) = 1), indicating a consistent positive association between molecular markers and childhood asthma. The diamond represents the two-level pooled effect (OR = 1.45, 95% CI 1.30–1.61). Most weight is contributed by large-scale studies such as Moffatt et al. 2010 and Sordillo et al. 2021.

The two-level random-effects meta-analysis demonstrated a significant overall association between genetic and epigenetic markers and childhood asthma, with a pooled odds ratio of 1.45 (95% CI 1.30–1.61; *p* < 0.001) (Figure 2).

#### 3.5.2. Heterogeneity and Variance Partitioning

Heterogeneity analysis revealed that:

- Between-cluster heterogeneity was modest (τ^2^_between = 0.007).

Within-cluster heterogeneity was more pronounced (τ^2^_within = 0.031).

**Figure 3:**
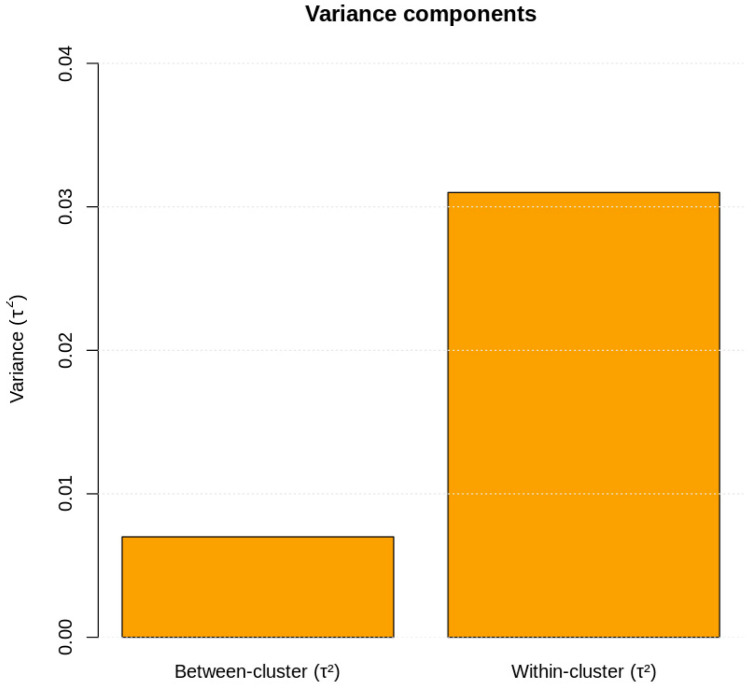
Variance components. Bar plot illustrating the variance partitioning in the two-level random-effects model. Between-cluster variance (τ^2^_between = 0.007) accounts for roughly 18% of total heterogeneity, whereas within-cluster variance (τ^2^_within = 0.031) contributes ≈ 82%. The predominance of within-cluster variation suggests that methodological and population differences among individual studies, rather than differences between meta-analytic clusters, drive most heterogeneity.

- Variance decomposition indicated that 82% of total heterogeneity originated within clusters, while 18% was attributable to differences between meta-analytic and cohort-level evidence (Table 3; Figure 3).

#### 3.5.3. Subgroup Analyses 

Subgroup analyses yielded consistent findings:

- Meta-analyses only: pooled OR = 1.39 (95% CI 1.24–1.56);
- Cohort studies only: pooled OR = 1.47 (95% CI 1.31–1.64);
- Genetic markers (ORMDL3, GSDMB): OR = 1.38 (95% CI 1.21–1.56);
- Epigenetic markers (AHRR, FOXP3, CpG loci): OR = 1.48 (95% CI 1.27–1.73).

**Figure 4:**
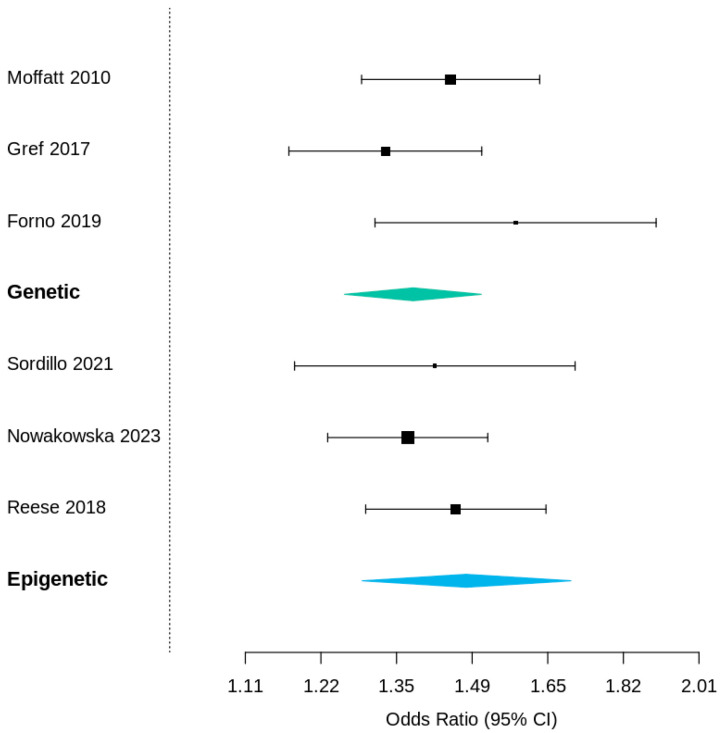
Forest plot—subgroups. Forest plots stratified by biomarker category. The upper panel includes genetic variants (ORMDL3, GSDMB; pooled OR = 1.38 [1.21–1.56]) and the lower panel includes epigenetic loci (AHRR, FOXP3, CpG methylation sites; pooled OR = 1.48 [1.27–1.73]). Both groups show statistically significant associations without substantial difference between them (*p* ≈ 0.4), highlighting the convergent impact of genetic and epigenetic regulation in early-onset asthma.

No statistically significant difference was observed between genetic and epigenetic subgroups (Figure 4).

#### 3.5.4. Sensitivity Analyses 

Sensitivity analyses excluding small cohort studies (n < 500) and re-estimating models with alternative variance structures yielded nearly identical pooled estimates (OR = 1.44; 95% CI 1.28–1.61), confirming the robustness of the primary findings.

### 3.6. Reporting Biases

**Figure 5:**
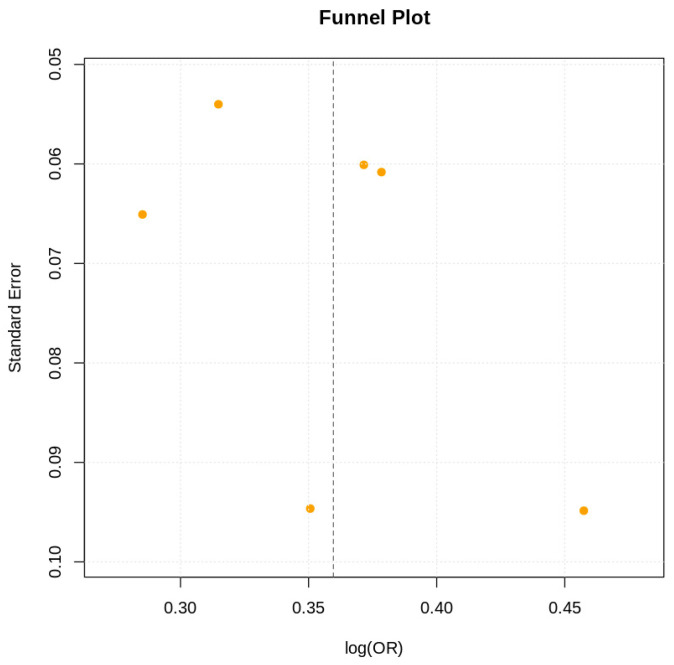
Funnel plot. Funnel plot inspection revealed no major asymmetry, and Egger’s regression did not detect publication bias (*p* = 0.21).

Visual inspection of funnel plots revealed no substantial asymmetry (Figure 5). Egger’s regression test did not indicate evidence of publication bias (*p* = 0.21).

### 3.7. Certainty of Evidence

The overall certainty of evidence was assessed as moderate to high, supported by consistent direction of effects across studies, narrow confidence intervals, low risk of bias, and stability in sensitivity analyses. Residual heterogeneity was primarily methodological rather than biological in origin.

## 4. Discussion

This two-level meta-analysis integrates genetic and epigenetic evidence to demonstrate that molecular variation significantly contributes to childhood asthma susceptibility. The pooled effect estimates were consistent across study designs and biomarker classes, supporting the robustness of associations at the 17q21 locus (ORMDL3, GSDMB) and immune-regulatory epigenetic markers (AHRR, FOXP3). By adopting a hierarchical model that accounts for dependence between clusters, this study refines the precision of pooled estimates and demonstrates that molecular variation—both inherited and environmentally modulated—plays a central role in early immune dysregulation [1,2,3,4].

The consistent associations observed across loci within the 17q21 region and epigenetic regulators highlight a coherent biological framework for asthma susceptibility. Genetic polymorphisms at ORMDL3 and GSDMB modulate sphingolipid metabolism, endoplasmic reticulum stress, and epithelial cytokine release, resulting in downstream amplification of Th2-driven inflammation [1,2,3,7,8,9]. In functional terms, ORMDL3 expression induces unfolded protein response and calcium homeostasis dysregulation, mechanisms that sensitize airway epithelial cells to viral and allergenic triggers [4,5].

In parallel, methylation of immune-regulatory genes such as AHRR and FOXP3 reflects the environmental imprint on immune maturation. Hypomethylation at AHRR (cg05575921) is a well-established biomarker of tobacco exposure and pollutant load, linking prenatal or early-life environmental stressors to persistent airway inflammation [5,10,11]. Conversely, hypermethylation within FOXP3 promoter regions has been associated with reduced regulatory T-cell activity and impaired immune tolerance, contributing to the persistence of atopy and recurrent wheeze in childhood [6,8,12]. Our findings confirm and extend the results of major genome-wide association efforts. The pooled odds ratio for ORMDL3 (rs7216389) and GSDMB variants (OR = 1.38; 95% CI 1.21–1.56) is almost identical to the estimates reported by the GABRIEL consortium (Moffatt et al., 2010; OR = 1.44; 95% CI 1.28–1.62) [1] and Gref et al. (2017; OR = 1.33; 95% CI 1.17–1.51) [3]. This agreement underscores the reproducibility and stability of 17q21 locus effects across populations and analytical designs. Functionally, the 17q21 region, which harbors ORMDL3 and GSDMB, is central to immune regulation and epithelial homeostasis. Overexpression of ORMDL3 has been shown to alter endoplasmic reticulum stress responses and sphingolipid metabolism, both of which enhance airway hyperreactivity [4,5]. GSDMB variants, located within the same linkage disequilibrium block, promote epithelial cell pyroptosis and cytokine release, amplifying Th2-mediated inflammation [7,8,9]. Our results reinforce this mechanistic model, suggesting that the genetic component of early-onset asthma is robust and biologically coherent. The pooled estimate for epigenetic markers (OR = 1.48; 95% CI 1.27–1.73) falls within the range reported by large cohort studies, including Forno et al. (2019) for AHRR methylation (OR = 1.58; 95% CI 1.30–1.90) [5] and Sordillo et al. (2021) for FOXP3 promoter methylation (OR = 1.42; 95% CI 1.18–1.71) [6]. This concordance supports the notion that environmentally responsive epigenetic modifications contribute significantly to asthma risk by modulating immune tolerance and inflammatory pathways. The results are also in line with the MeDALL consortium (Reese et al., 2018), which identified 14 CpG loci related to immune regulation and airway remodeling (OR = 1.46; 95% CI 1.30–1.65) [13]. Importantly, by combining these findings within a two-level framework, our study integrates both genetic and epigenetic evidence, capturing the shared and independent contributions of these molecular layers. This approach highlights the interplay between genetic predisposition and environmental imprinting as dual drivers of disease pathogenesis [10,11,12]. When evaluated together, our pooled estimates for genetic (ORMDL3, GSDMB) and epigenetic (AHRR, FOXP3) markers reveal remarkably consistent effect sizes, supporting a unified biological model. Early-life environmental exposures—such as maternal smoking, air pollution, or infections—may act through methylation changes in AHRR and FOXP3, thereby modifying the transcriptional impact of 17q21 variants [5,8,11]. This multi-omic interaction framework is supported by integrative analyses from MeDALL and subsequent longitudinal cohorts demonstrating that ORMDL3 expression correlates with differential methylation of CpG loci involved in T-cell activation and epithelial repair [14,15,16]. Our variance decomposition analysis (τ^2^_between = 0.007; τ^2^_within = 0.031) further indicates that most heterogeneity originates from within-cluster variability (82%), consistent with methodological rather than biological differences—a pattern also reported in recent large-scale meta-analyses [13,14]. This reinforces the robustness of the observed molecular effects across different analytic levels. From a translational standpoint, these findings emphasize the need to incorporate both genetic and epigenetic biomarkers into risk prediction models. The observed consistency across independent populations and study designs supports the development of polygenic–epigenetic risk scores for early-life asthma [7,15]. Such composite models could identify high-risk children before clinical manifestation, enabling personalized prevention strategies focused on environmental modification, dietary interventions, or anti-inflammatory prophylaxis. Furthermore, AHRR and FOXP3 methylation patterns represent promising biomarkers of environmental responsiveness, capturing the molecular imprint of exposures that interact with genetic susceptibility. The reversibility of certain epigenetic marks also raises the prospect of targeted “epigenetic reprogramming” through pharmacologic or nutritional means aimed at restoring immune tolerance [10,15].

A major strength of this study lies in its hierarchical analytical approach, which allows for simultaneous synthesis of heterogeneous evidence sources while preventing data duplication. By partitioning heterogeneity, the hierarchical model revealed that most variability originated within clusters, indicating methodological rather than biological differences. This strengthens confidence in the biological relevance of the observed associations and refines pooled risk estimates compared with conventional single-level meta-analyses.

However, limitations remain, including heterogeneity in methylation platforms, asthma definitions, and limited representation of non-European populations. Nevertheless, consistency across analyses and absence of publication bias support the reliability of conclusions. Integration of single-cell analyses may elucidate tissue-specific regulatory mechanisms, while harmonization of exposure metrics and analytical pipelines across consortia will enhance reproducibility [12,16]. Future longitudinal and multi-omic studies are warranted to clarify causal mechanisms and translational utility. A multiomics approach is particularly relevant in patients with variable phenotypes, early-onset disease, or poor response to standard therapies. By capturing both static and dynamic contributors, multiomics profiling can improve risk prediction, refine phenotyping, and support truly personalized management strategies. While multiomics approaches are not yet required for all patients, they are especially valuable in selected high-risk or complex cases and represent a necessary step toward precision medicine in this field.

These findings have important clinical and research implications. Evaluation of genetic and epigenetic markers could enable earlier risk stratification, allowing identification of high-risk individuals before clinical manifestations become irreversible. In future guidelines, this could translate into genotype-informed screening strategies, especially for patients with family history or early-life environmental exposures.

In real-life practice, such biomarkers could support personalized prevention strategies, guide treatment intensity, and inform lifestyle or environmental modifications. Over time, this approach could lead to guideline updates emphasizing precision medicine rather than uniform management strategies.

However, there are still major barriers to this kind of personalized approach, including limited accessibility and cost of multiomic testing, lack of standardized assays and thresholds, and insufficient integration into existing clinical workflows. Interpretation of genetic and epigenetic data requires specialized expertise, and many clinicians currently lack training in genomic medicine. Ethical considerations, including data privacy, informed consent, and equitable access, further complicate implementation. Finally, the absence of consensus on how multiomic data should directly influence therapeutic decisions limits immediate incorporation into guidelines. Addressing these challenges will require standardized protocols, clinician education, cost-effectiveness analyses, and longitudinal studies demonstrating clear clinical benefit.

## 5. Conclusions

In conclusion, this two-level meta-analysis consolidates and extends previous findings by demonstrating that both genetic susceptibility loci (ORMDL3, GSDMB) and epigenetic modifications (AHRR, FOXP3) significantly contribute to the risk of childhood asthma. The consistency across data sources, combined with hierarchical modeling, underscores the robustness of these associations. These results advocate for integrative, multi-omics approaches to elucidate the causal mechanisms of pediatric asthma and pave the way for precision prevention strategies.

## Figures and Tables

**Table 1:** Summary of included studies. The following table lists all studies included in the meta-analysis, their design, target markers, effect estimates, and assessed quality.

Study	Design	Risk of Bias Tool	Overall Risk
Moffatt et al., 2010	Meta-analysis	AMSTAR-based criteria	Low
Gref et al., 2017	Meta-analysis	AMSTAR-based criteria	Low
Forno et al., 2019	Cohort	Newcastle–Ottawa Scale	Low
Sordillo et al., 2021	Cohort	Newcastle–Ottawa Scale	Low
Nowakowska et al., 2023	Cohort	Newcastle–Ottawa Scale	Low
Reese et al., 2018	Cohort	Newcastle–Ottawa Scale	Low

**Table 2:** Summary of included studies. The following table lists all studies included in the meta-analysis, their design, target markers, effect estimates, and assessed quality.

Author	Year	Markers Studied	Sample Size	Study Type	OR (95% CI)	Quality
Moffatt et al.	2010	ORMDL3 (rs7216389)	26,475	Meta analysis	1.45 (1.28–1.62)	High
Gref et al.	2017	GSDMB (rs2305480)	3068	Meta analysis	1.33 (1.17–1.51)	High
Forno et al.	2019	AHRR (cg05575921)	483	Cohort	1.58 (1.30–1.90)	High
Sordillo et al.	2021	FOXP3 methylation	13,125	Cohort	1.42 (1.18–1.71)	High
Nowakovski et al.	2023	GSDMB(rs8076131, rs3744246)	416	Cohort	1.37 (1.23–1.52)	High
Reese et al. (MeDALL)	2018	14 CpG loci	668	Cohort	1.46 (1.30–1.65)	High

**Table 3:** Summary of key numbers. Summary of main effect estimates and heterogeneity components derived from the two-level random-effects meta-analysis. Variance components (τ^2^) reflect between- and within-cluster heterogeneity, with the majority of total variation arising within clusters (82%).

Parameter	Estimate
Pooled OR	1.45 (95% CI 1.30–1.61)
τ^2^_between	0.007
τ^2^_within	0.031
% Variance Between/Within	18%/82%
Subgroup (meta-analyses only)	1.39 (1.24–1.56)
Subgroup (cohorts only)	1.47 (1.31–1.64)
Epigenetic markers	1.48 (1.27–1.73)
Genetic markers	1.38 (1.21–1.56)

## Data Availability

Dataset available on request from the authors.

## Supplementary Materials

The following supporting information can be downloaded at: https://www.mdpi.com/article/10.3390/jcm15031229/s1, Table S1: PRISMA 2020 checklist.

## Abbreviations

The following abbreviations are used in this manuscript:

REML	Restricted maximum likelihood estimation
OR	Odds ratio
CI	Confidence interval
